# Once we have it, will we use it? A European survey on willingness to be vaccinated against COVID-19

**DOI:** 10.1007/s10198-020-01208-6

**Published:** 2020-06-26

**Authors:** Sebastian Neumann-Böhme, Nirosha Elsem Varghese, Iryna Sabat, Pedro Pita Barros, Werner Brouwer, Job van Exel, Jonas Schreyögg, Tom Stargardt

**Affiliations:** 1 Erasmus School of Health Policy and Management, Erasmus University Rotterdam, Rotterdam, The Netherlands; 2 Centre for Research on Health and Social Care Management, CERGAS, Bocconi University, Milan, Italy; 3 Nova School of Business and Economics, Carcavelos, Portugal; 4 Erasmus School of Economics, Erasmus University Rotterdam, Rotterdam, The Netherlands; 5 Hamburg Center for Health Economics, University of Hamburg, Hamburg, Germany

**Keywords:** I10, I18

## Introduction

While the focus of attention currently is on developing a vaccine against the Coronavirus SARS-CoV-2 to protect against the disease COVID-19, policymakers should prepare for the next challenge: uptake of the vaccine among the public. Having a vaccine does not automatically imply it will be used. Compliance with the anti-H1N1 vaccine during the 2009 influenza pandemic, for instance, was low [1], and in the decade since, vaccination rates have remained an issue of concern [2] while vaccination hesitancy has become more prevalent, leading to increases in disease outbreaks in multiple countries [3]. It is, therefore, important to understand whether or not people are willing to be vaccinated against COVID-19, as this can have large consequences for the success a vaccination programme—with potentially large health and economic consequences. In this editorial, we provide some first insights into this willingness to be vaccinated, based on a multi-country European study [4], which hopefully result in more attention for this important issue.

## A vaccine against COVID-19

On April 26, the WHO counted seven COVID-19 candidate vaccines in the clinical evaluation phase and 82 more in the preclinical evaluation phase [5]. This underlines the unprecedented current efforts worldwide to find an effective vaccine against the Coronavirus SARS-CoV-2. Some expect that first vaccines may become available under emergency use protocols as soon as early 2021, given the speed and scale of research and development efforts globally, while others argue it will take longer [6–8]. In both cases, the development phase should be followed by large-scale vaccination programmes to attain herd immunity [9]. That way, we can protect the lives of the most vulnerable people and reduce the social and economic burden of the current crisis.

Vaccination programmes can lead to herd immunity without requiring a large proportion of the population to be infected. The latter is mostly seen as an undesirable option, given the potentially high numbers of deaths as a result of infection. Especially so, if the health systems are overwhelmed by a large number of patients with severe COVID-19 symptoms [10]. Herd immunity through vaccination, however, requires a sufficient proportion of the population to be vaccinated. While vaccination is widely recognised as an effective way to reduce or eliminate the burden of infectious diseases by health authorities and the medical community [11], its effectiveness also depends on the individual willingness to be vaccinated. This willingness could be negatively affected by doubts and worries that exist in the population about the safety and appropriateness of vaccines. This is sometimes labelled vaccine hesitancy [12]. If too many individuals hesitate about being vaccinated, herd immunity may not be reached. Besides objective trade-offs of costs and benefits of a vaccine, risk attitude, pro-social considerations, and misinformation or misperceptions about a vaccine may play a role in this [2, 13, 14].

At present, it is unclear whether a sufficient proportion of the population would decide to get vaccinated when a vaccine becomes available. In the EU, vaccine delays and refusals are contributing to declining immunisation rates in several countries and lead to increases in disease outbreaks [3]. Hence, and the question is whether enough Europeans trust the effectiveness and safety of vaccines and the healthcare system that delivers them [15].

## Willingness to be vaccinated

To shed more light on the issue of willingness to be vaccinated, we investigated people attitudes about vaccination against COVID-19 in an online survey among representative samples of the population (in terms of region, gender, age group and education) in seven European countries (*N* = 7.662). The sample consisted of about 1.000 respondents per country, and an additional 500 from the highly affected region Lombardy, since we expected that results might differ from the rest of Italy. In this first wave of the data collection, respondents were inquired about worries and beliefs about COVID-19, as well as attitudes about vaccination and their willingness to be vaccinated between 2 and 15 April 2020 [4]. In this editorial, we provide some first insights into the findings, to stimulate further research and policy in this area.

**Fig. 1:**
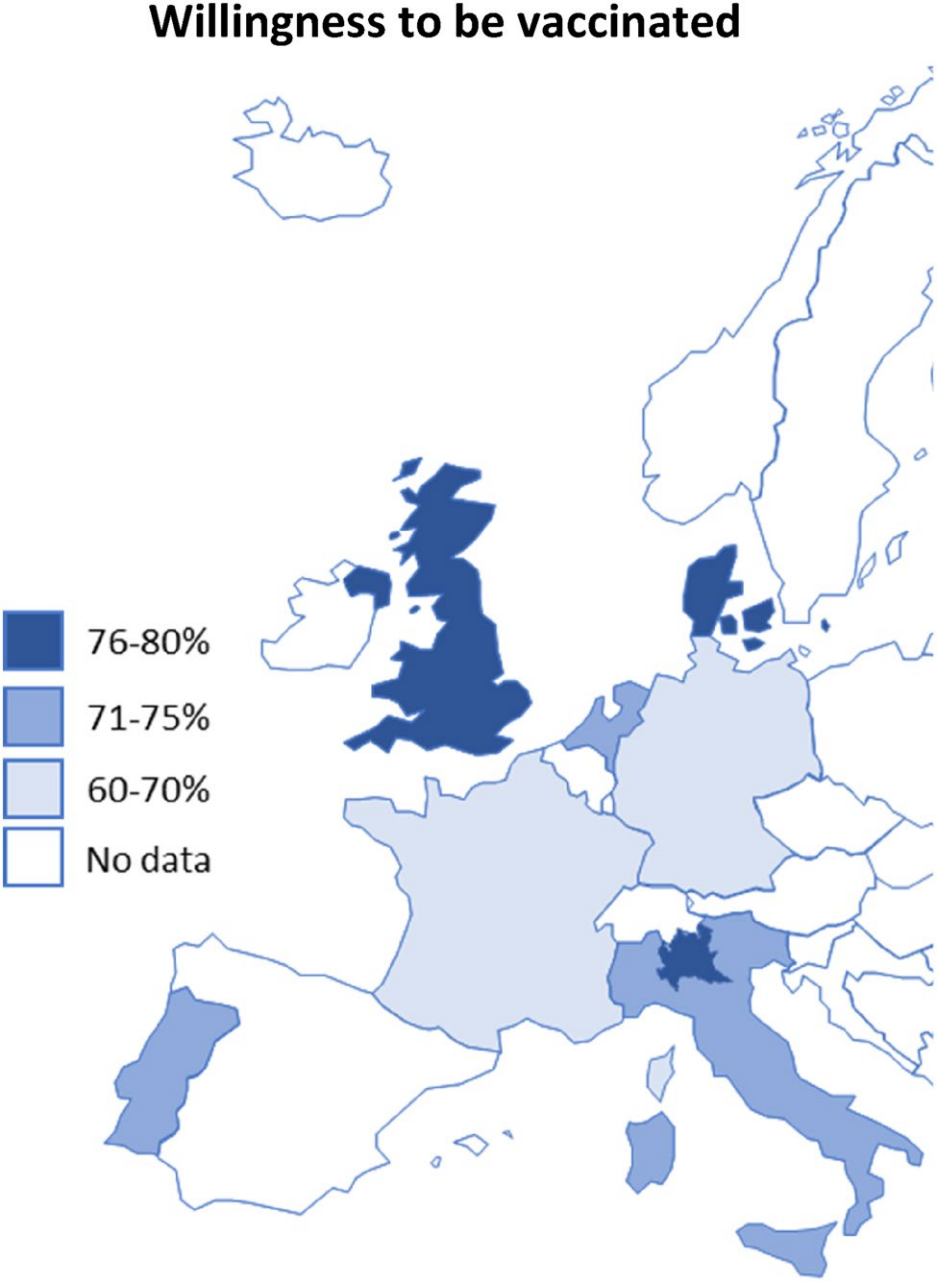
Proportion of respondents who stated they would be willing to be vaccinated against the novel coronavirus per country

**Fig. 2:**
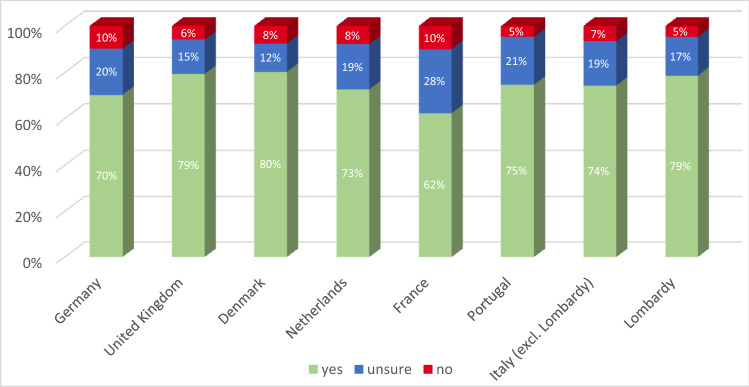
Willingness to be vaccinated against the coronavirus by country

In total, 73.9% of the 7664 participants from Denmark, France, Germany, Italy, Portugal, the Netherlands, and the UK stated that they would be willing to get vaccinated against COVID-19 if a vaccine would be available. A further 18.9% of respondents stated that they were not sure, and 7.2% stated that they do not want to get vaccinated. As shown in Figs. 1 and 2, the willingness ranged from 62% in France to approx. 80% in Denmark and the UK. The largest proportions of the population opposed to a COVID-19 vaccination were observed in Germany (10%) and France (10%), while France also has the largest group of people who were unsure about getting vaccinated (28%).

**Fig. 3:**
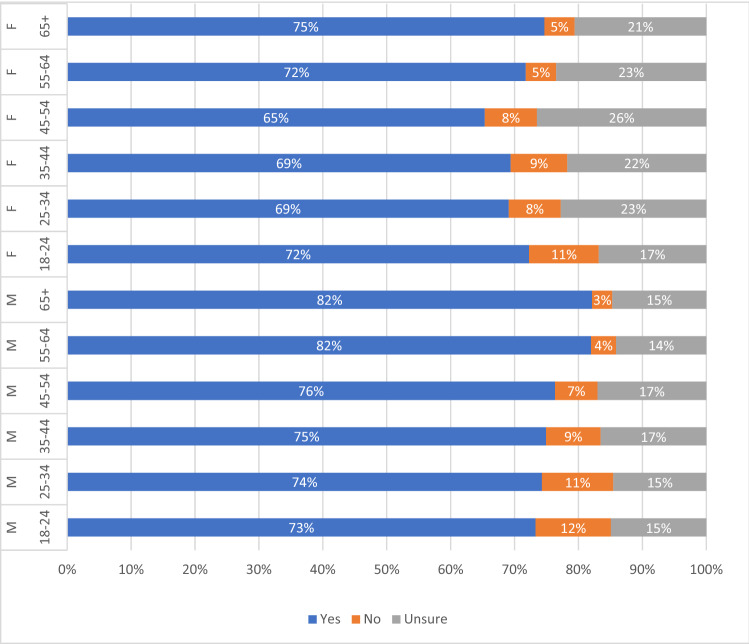
Willingness to be vaccinated against COVID-19 by age group and gender

Looking closer, we found considerable differences in willingness to get vaccinated across genders and age groups (Fig. 3). A significantly higher proportion of men were willing to get vaccinated (77.94%, Chi-squared, *p *< 0.001) than women (70.15%). The willingness to be vaccinated is largest among men above the age of 55, while uncertainty ranged between 14 and 17% across all age groups. Males who were unwilling to get vaccinated tended to be younger with the largest share of 12% among the 18–24 year olds. Similarly, the trend for women who were unwilling to vaccinate seems also to follow the age categories. The uncertainty among women was higher in all age groups and largest for women between the ages of 45 and 54 (26%).

One might argue that the group who is currently unsure about getting a vaccine may be the most relevant. These are the people who potentially can be persuaded more easily to get vaccinated to achieve herd immunity. Based on our results, these efforts could best be aimed at persons below the age of 55 and at females in general, where the willingness is lower.

**Fig. 4:**
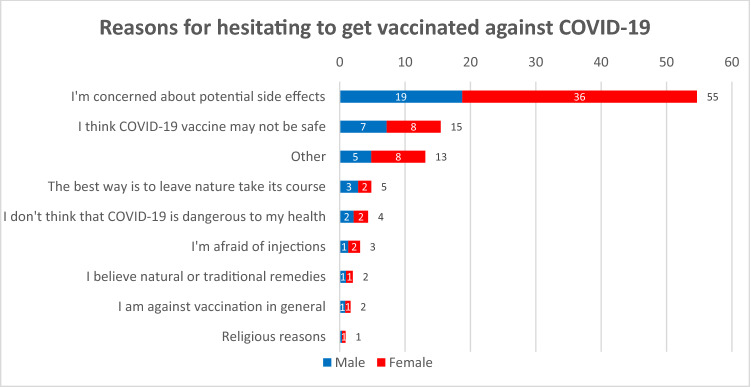
Reasons given by people who were unsure if they would like to be vaccinated against COVID-19 in percent, *N* = 1451

We asked respondents who were unsure about being vaccinated about their main reasons (Fig. 4). More than half (55%) said they were concerned about potential side effects of a vaccine, although this concern was more frequent among women (36%) than men (19%). Around 15% of respondents stated that a vaccine might not be safe, with no notable differences between genders. These findings are in the literature on frequent reasons for vaccine hesitancy [15]. Looking at the open text explanations given to the category “other”, we saw that a common concern seems to be that a COVID-19 vaccine might be experimental, without any studies on side effects, and that the vaccine may not be safe for specific groups, such as for pregnant woman, people with pre-existing conditions like MS, allergic persons etc.

This finding highlights that while the current focus seems to be on developing a vaccine about ten times faster than usual [7], the public should also be reassured that any vaccine which becomes available that quickly is safe and effective. Otherwise, there is a risk to lose the public trust in the particular vaccine, and coronavirus vaccination altogether [16], potentially compromising herd immunity.

**Fig. 5:**
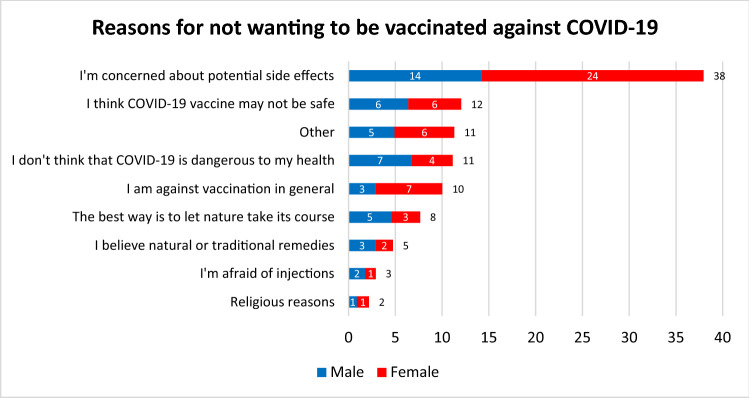
Reasons for not getting vaccinated against COVID-19 in percent, *N* = 548

We find a similar trend regarding the most frequently mentioned reasons and the gender differences for the concerns about side effects among those who were not willing to get vaccinated (Fig. 5). Notable gender differences could also be observed among those respondents who stated that they think COVID-19 is not dangerous to their health (11%), comprised of almost twice as many men (7%) than women (4%). Furthermore, we see that an overall rejection of vaccination was more than twice as common among women (7%) than among men (3%). When looking at the open text answers of respondents who choose other reasons (11%), we found not only concerns about safety but also comments about conspiracy theories and a general rejection of vaccines.

## Increasing willingness to be vaccinated

The literature suggests multiple steps that could be taken by policymakers to decrease vaccine hesitancy and convince doubters to get vaccinated after all. One approach for vaccine advocacy suggests “vaccine adoption = access + acceptance” [17]. Looking at access, it is essential to translate the willingness to be vaccinated into actual vaccination decisions. Our study measured the intention to vaccinate; this rate might differ from actual vaccination uptake (vaccination decision) depending on potential constraints, such as the price of the vaccine and the ease of access of vaccination sites. Vaccines should thus be available in a timely manner and an easily accessible way to have as little attrition as possible [12]. In the case of the coronavirus vaccine, access will prove quite challenging since, at the early stages of availability, the demand for this vaccine worldwide will be much greater than the (short-term) production capacities. Currently, about 5 billion doses of vaccine are produced yearly worldwide, of which 30% are seasonal flu vaccines [18]. So even when a vaccine becomes available, access to it will probably be limited in the short run. Therefore, policymakers need to prepare how access can be organised equitably and effectively.

Our results on acceptability suggest that substantial gains could be made among the sizeable proportion of the population (i.e. 18.9%) that is unsure whether they want to get vaccinated. If this group needs to be convinced to be vaccinated to get to herd immunity, clear communication about safety, and potential side effects of the vaccine is especially important. This could help to stimulate the hesitant part of European citizens to get vaccinated after all.

This is especially important since it is unclear whether the group of people who are willing to be vaccinated in itself is large enough to achieve herd immunity. The basic reproduction number $R_{0}$ shows the transmission potential of diseases [19], i.e. to how many people the infection is expected to be passed on by one infected individual in a fully susceptible population, on average. The herd immunity threshold describes the proportion of the population that needs to be immune, so that the infectious disease is stable (*R* = 1) and is calculated as [20]:

$$ {\text{Herd immunity threshold}} = 1 - \frac{1}{{R_{0} }}. $$

This means that the higher the basic reproductive number $R_{0}$ is, the higher the herd immunity threshold becomes. A recent study estimated a COVID-19 $R_{0}$ of around 3.87 for Europe [21], implying a herd immunity threshold for Europe of 74%. For the US, it was estimated at around 3.45, implying a herd immunity threshold of 71% [22]; while, a recent study argues these values may be lower if there is heterogeneity in the individual susceptibility to the virus [23]. Of course, these estimates are uncertain, but comparing this 71–74% threshold range with our results indicates that the current willingness levels in France, Germany and the Netherlands, in particular, may prove insufficient to reach this threshold.

Our survey highlighted important differences between citizens from European countries in terms of willingness to be vaccinated against COVID-19. The levels do not follow trends that we see in other vaccination rates, e.g. against measles, which are generally higher, but in most countries below the recommended 95% threshold [24].

Understanding which groups in the population are not willing to be vaccinated and why remains vital for the design of policy responses to vaccination hesitancy. One of the avenues to explore could be to emphasise the social benefits of vaccination more strongly so that they weigh the public health dimension more heavily in their decision whether to vaccinate [13]. A recent study, for example, found that people are more willing to get vaccinated when they were informed that this would protect others who are willing but unable to get vaccinated themselves [25]. Consequently, one of the communication strategies could be to emphasise how vaccination against COVID-19 helps to protect vulnerable members of society. Furthermore, the distribution of vaccinated individuals in the population matters. Pockets of non-vaccinated groups could be highly problematic even when overall vaccination rates are high. Unvaccinated individuals may be more often in contact with other unvaccinated individuals than with vaccinated ones [26]. Examples of measles outbreaks in the Netherlands [27] and the US [28], for instance, show that outbreaks in particular communities may even occur if overall vaccination rates are high, and highlight the role of religious communities and travellers in this context.

Alternative strategies range from restrictive measures against those who chose not to be vaccinated to mandatory vaccination schemes for certain target groups or the whole population. Experimental evidence suggests that individuals under specific conditions may be willing to support mandatory vaccination policies, but this support seems very sensitive to adverse events [29]. Such a policy may be less appropriate in the context of COVID-19.

### Beyond finding a vaccine

Our findings highlight that considerable policy effort may be required to come from having a vaccine to adequate vaccination rates, especially in some countries. Targeting those in the population who are currently hesitant seems most promising and cost-effective, but this requires convincing evidence and clear communication on the safety and effectiveness of the vaccine. This may be at odds with the current push for having a vaccine available as soon as possible. A campaign emphasising the social benefits of vaccination could increase the willingness to be vaccinated among those amenable to such pro-social motives. Finally, a sizeable proportion of the population indicates not to be open to vaccination. This group may remain at risk of spreading the virus and contracting the disease, even after herd immunity has been achieved. Concluding, improving our understanding of vaccination hesitancy in the context of COVID-19, as well as finding and using policies to overcome it, may be as important as discovering a safe and effective vaccine.
